# Lymph Node Regression Improves Prognostic Stratification in Esophageal Squamous Cell Carcinoma Receiving Neoadjuvant Chemoimmunotherapy: A Multicenter Retrospective Cohort Study

**DOI:** 10.1002/mco2.71075

**Published:** 2026-10-21

**Authors:** Jian Zheng, Wenbin Shen, Jianzhong Cao, Linrui Gao, Likun Zan, Tian Zhang, Lei Gong, Xiaofeng Duan, Xiaobin Wang, Yueqin Wu, Peng Tang, Hongjing Jiang, Qingsong Pang, Gang Zhao, Wencheng Zhang, Cihui Yan

**Affiliations:** ^1^ Department of Radiation Oncology Tianjin Medical University Cancer Institute & Hospital National Clinical Research Center for Cancer State Key Laboratory of Druggability Evaluation and Systematic Translational Medicine Key Laboratory of Cancer Prevention and Therapy Tianjin's Clinical Research Center for Cancer Tianjin China; ^2^ Department of Radiotherapy The Fourth Hospital of Hebei Medical University Shijiazhuang China; ^3^ Department of Radiotherapy Shanxi Province Cancer Hospital Shanxi Hospital Affiliated to Cancer Hospital Chinese Academy of Medical Sciences Cancer Hospital Affiliated to Shanxi Medical University Taiyuan China; ^4^ Department of Pathology Shanxi Province Cancer Hospital/ Shanxi Hospital Affiliated to Cancer Hospital Chinese Academy of Medical Sciences Cancer Hospital Affiliated to Shanxi Medical University Taiyuan China; ^5^ Department of Esophageal Surgery Tianjin Medical University Cancer Institute and Hospital National Clinical Research Center of Cancer Key Laboratory of Cancer Prevention and Therapy of Tianjin Tianjin's Clinical Research Center for Cancer Hexi Tianjin China; ^6^ Department of Minimally Invasive Esophageal Surgery Tianjin Medical University Cancer Hospital and Institute National Clinical Research Center for Cancer Key Laboratory of Cancer Prevention and Therapy Tianjin China; ^7^ Department of Pathology Tianjin Medical University Cancer Institute & Hospital National Clinical Research Center for Cancer Key Laboratory of Cancer Prevention and Therapy Tianjin's Clinical Research Center for Cancer Tianjin China; ^8^ Department of Immunology Tianjin Medical University Cancer Institute & Hospital National Clinical Research Center for Cancer State Key Laboratory of Druggability Evaluation and Systematic Translational Medicine Key Laboratory of Cancer Immunology and Biotherapy Tianjin's Clinical Research Center for Cancer Tianjin China

**Keywords:** esophageal cancer, neoadjuvant therapy, immunotherapy, lymph node regression

## Abstract

Lymph nodes (LNs), principal sites of antitumor immunity, may undergo immune‐mediated regression; whether neoadjuvant chemoimmunotherapy (NCIT)‐induced regression indicates prognosis in esophageal squamous cell carcinoma (ESCC) remains unclear. We aimed to establish and validate a lymph node regression grade (LNRG) system for prognostic stratification. This multicenter, retrospective cohort study analyzed 965 patients receiving PD‐1‐based NCIT and surgery at three Chinese tertiary cancer centers. LNRG was the ratio of summed viable tumor area to summed bed area (residual viable plus regressed preexisting tumor) across all dissected LNs. Negative LNs were assigned LNRG 0. Survival and predictive performance were assessed using Kaplan–Meier/Cox regression, time‐dependent area under the curve, Brier score, net reclassification improvement, and integrated discrimination improvement. Overall, 28,882 LNs were evaluated; patients were classified as LNRG 0–4 in 385 (39.9%), 150 (15.5%), 26 (2.7%), 141 (14.6%), and 263 (27.3%), respectively. LNRG independently predicted disease‐free and overall survival, with Grades 1–2 conferring the most favorable outcomes and Grade 4 the poorest. A random survival forest model incorporating LNRG, tumor regression grade, ypAJCC stage, and perineural invasion predicted 2‐year disease‐free survival in training and external validation cohorts. LNRG is a novel, independent prognostic indicator after NCIT.

## Graphical abstract

Pathological lymph node regression grading predicts survival after neoadjuvant chemoimmunotherapy. (A) Schematic illustration of the novel five‐tier lymph node regression grading (LNRG) system and representative histopathological images. LNRG 1–4 were defined as follows: LNRG 1, no viable tumor cells observed within the tumor bed; LNRG 2, viable tumor occupying < 10% of the tumor bed area; LNRG 3, viable tumor occupying 10%–50% of the tumor bed area; and LNRG 4, viable tumor occupying > 50% of the tumor bed area. LNRG 0, negative lymph nodes.For patients with multiple metastatic lymph nodes, the overall LNRG was determined according to the proportion of the total viable tumor area to the total tumor bed area across all metastatic lymph nodes.(B) Association of LNRG with disease‐free survival. CI, confidence interval; HR, hazard ratio; LNRG, lymph node regression grade.

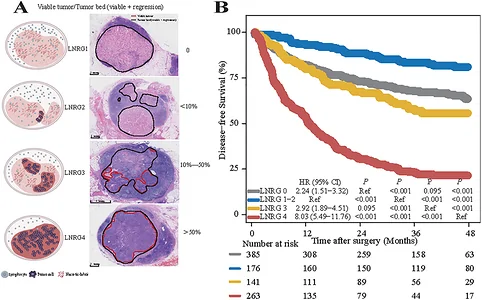

## 1 Introduction

Esophageal cancer (EC) ranks eighth globally in terms of cancer incidence, with esophageal squamous cell carcinoma (ESCC) being the predominant histological subtype in Asia [1]. Recent clinical studies have reported that neoadjuvant chemoimmunotherapy (NCIT) achieves pathologic complete response (pCR) rates ranging from 16.7% to 55.6%, with manageable treatment‐related toxicities [2, 3]. Accordingly, NCIT has emerged as a potential strategy for ESCC. Nevertheless, a large‐scale retrospective study demonstrated that the 2‐year recurrence rate remains 23.7% among patients with ESCC receiving NCIT [4], highlighting the need for more precise stratification of recurrence risk in this setting.

Pathological tumor regression grading (TRG) is the most widely used metric for evaluating treatment response after neoadjuvant therapy and provides important information on outcomes in patients with ESCC undergoing NCIT [5]. However, TRG primarily reflects regression in the primary tumor (PT) and does not capture immune‐mediated changes in metastatic lymph nodes (LNs). Accumulating studies suggest that LN‐derived stem‐like CD8+ T cells, rather than tumor‐resident CD8+ T cells, are key drivers of antitumor immunity [6, 7]. Consistent with this biology, clinical studies have shown that the degree of pathological LN regression, independent of other prognostic variables such as TRG, is associated with improved survival and reduced recurrence in patients with EC treated with neoadjuvant chemotherapy (NCT) or chemoradiotherapy (NCRT) [8, 9]. To date, only a preliminary study has reported an association between LN regression and overall survival (OS) in patients with ESCC undergoing NCIT; however, this association did not demonstrate independent significance beyond TRG [10]. These limitations highlight the importance of establishing a standardized LN regression grading (LNRG) system to complement TRG in evaluating treatment outcomes in patients with ESCC receiving NCIT.

Based on a multicenter dataset, we herein established a novel LNRG system, which was identified as an independent prognostic indicator of outcomes in patients with ESCC receiving NCIT. Furthermore, by combining LNRG with ypAJCC stage, perineural invasion (PNI), and TRG, we developed and validated a clinically useful and interpretable machine learning model, Random survival forest (RSF), to predict early recurrence in ESCC patients receiving NCIT followed by surgery.

## 2 Results

### 2.1 Patient Characteristics

A total of 965 participants were included in this study from 7737 individuals initially screened (Figure 1). The eligible cohort had a median age of 63 years (IQR, 57–67 years), and the majority were men (83.73%). According to the eighth AJCC clinical stages, 7 patients were staged as Stage I, 300 as Stage II, 529 as Stage III, and 129 as Stage IV. The baseline and pathological characteristics of the study participants are summarized in Table 1.

**FIGURE 1:**
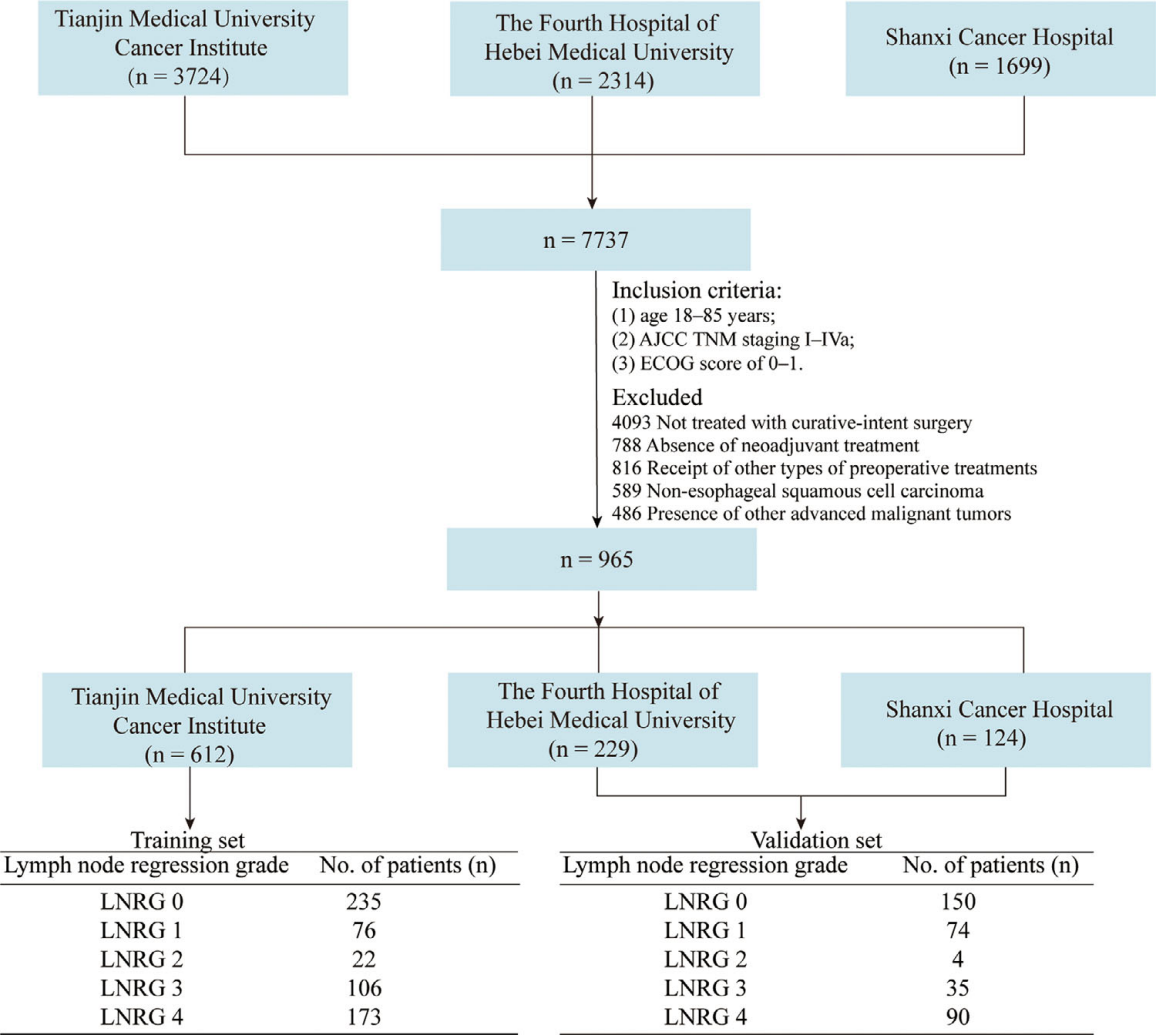
Flowchart of the study. AJCC, American Joint Committee on Cancer; ECOG, Eastern Cooperative Oncology Group; LNRG, lymph node regression grade.

**TABLE 1:** Baseline clinical and pathological characteristics and lymph node regression grading (*N* = 965).

	Overall No. (%)	LNRG0 No. (%)	LNRG1 —2 No. (%)	LNRG3 No. (%)	LNRG4 No. (%)	
Variable	(*n* = 965)	(*n = *385)	(*n = *176)	(*n = *141)	(*n = *263)	*p*
Age(year)						0.053
< 65	534 (55.34)	193 (50.13)	99 (56.25)	85 (60.28)	157 (59.70)	
≥ 65	431 (44.66)	192 (49.87)	77 (43.75)	56 (39.72)	106 (40.30)	
Gender						< 0.001
Male	808 (83.73)	297 (77.14)	149 (84.66)	129 (91.49)	233 (88.59)	
Female	157 (16.27)	88 (22.86)	27 (15.34)	12 (8.51)	30 (11.41)	
Smoking history						0.004
No	349 (36.17)	159 (41.30)	70 (39.77)	39 (27.66)	81 (30.80)	
Yes	616 (63.83)	226 (58.70)	106 (60.23)	102 (72.34)	182 (69.20)	
Alcohol misuse						< 0.001
No	438 (45.39)	202 (52.47)	88 (50.00)	48 (34.04)	100 (38.02)	
Yes	527 (54.61)	183 (47.53)	88 (50.00)	93 (65.96)	163 (61.98)	
Body mass index^a^						0.027
< 18	45 (4.66)	16 (4.16)	7 (3.98)	5 (3.55)	17 (6.46)	
18–24	617 (63.94)	243 (63.12)	102 (57.95)	93 (65.96)	179 (68.06)	
> 24	303 (31.40)	126 (32.73)	67 (38.07)	43 (30.50)	67 (25.48)	
Clinical T stage						0.511
1	16 (1.66)	7 (1.82)	5 (2.84)	1 (0.71)	3 (1.14)	
2	48 (4.97)	24 (6.23)	6 (3.41)	6 (4.26)	12 (4.56)	
3	817 (84.66)	317 (82.34)	150 (85.23)	118 (83.69)	232 (88.21)	
4	84 (8.70)	37 (9.61)	15 (8.52)	16 (11.35)	16 (6.08)	
Clinical *N* stage						< 0.001
0	292 (30.26)	174 (45.19)	73 (41.48)	8 (5.67)	37 (14.07)	
1	369 (38.24)	152 (39.48)	51 (28.98)	64 (45.39)	102 (38.78)	
2	256 (26.53)	52 (13.51)	45 (25.57)	59 (41.84)	100 (38.02)	
3	48 (4.97)	7 (1.82)	7 (3.98)	10 (7.09)	24 (9.13)	
AJCC clinical stage						< 0.001
I	7 (0.73)	1 (0.26)	3 (1.70)	1 (0.71)	2 (0.76)	
II	300 (31.09)	175 (45.45)	72 (40.91)	11 (7.80)	42 (15.97)	
III	529 (54.82)	166 (43.12)	80 (45.45)	103 (73.05)	180 (68.44)	
IV	129 (13.37)	43 (11.17)	21 (11.93)	26 (18.44)	39 (14.83)	
Tumor location						0.147
Proximal third	79 (8.19)	35 (9.09)	9 (5.11)	14 (9.93)	21 (7.98)	
Middle third	452 (46.84)	191 (49.61)	94 (53.41)	58 (41.13)	109 (41.44)	
Distal third	434 (44.97)	159 (41.30)	73 (41.48)	69 (48.94)	133 (50.57)	
ypT stage						< 0.001
0	261 (27.05)	138 (35.84)	77 (43.75)	19 (13.48)	27 (10.27)	
1	170 (17.62)	69 (17.92)	40 (22.73)	21 (14.89)	40 (15.21)	
2	182 (18.86)	79 (20.52)	20 (11.36)	34 (24.11)	49 (18.63)	
3	352 (36.48)	99 (25.71)	39 (22.16)	67 (47.52)	147 (55.89)	
ypN stage						< 0.001
0	535 (55.44)	385 (100.00)	150 (85.23)	0 (0.00)	0 (0.00)	
1	232 (24.04)	0 (0.00)	19 (10.80)	92 (65.25)	121 (46.01)	
2	144 (14.92)	0 (0.00)	5 (2.84)	42 (29.79)	97 (36.88)	
3	54 (5.60)	0 (0.00)	2 (1.14)	7 (4.96)	45 (17.11)	
AJCC pathologic stage						< 0.001
I	404 (41.87)	286 (74.29)	118 (67.05)	0 (0.00)	0 (0.00)	
II	131 (13.58)	99 (25.71)	32 (18.18)	0 (0.00)	0 (0.00)	
III	376 (38.96)	0 (0.00)	24 (13.64)	134 (95.04)	218 (82.89)	
IV	54 (5.60)	0 (0.00)	2 (1.14)	7 (4.96)	45 (17.11)	
Lymphovascular invasion						< 0.001
Negative	889 (92.12)	378 (98.18)	173 (98.30)	125 (88.65)	213 (80.99)	
Positive	76 (7.88)	7 (1.82)	3 (1.70)	16 (11.35)	50 (19.01)	
Perineural invasion						0.048
Negative	929 (96.27)	373 (96.88)	172 (97.73)	138 (97.87)	246 (93.54)	
Positive	36 (3.73)	12 (3.12)	4 (2.27)	3 (2.13)	17 (6.46)	

Abbreviations: AJCC, American Joint Committee on Cancer; HR, hazard ratio; LNRG, lymph node regression grade.

^a^ Body mass index was calculated as weight in kilograms divided by height in meters squared.

### 2.2 Average LNRG Classification in ESCC Patients Receiving NCIT

In the entire cohort of 965 patients, a total of 28,882 LNs were analyzed, with a median of 26 LNs examined per patient (range, 10–115). Negative LN regression (LNRG 0) was observed in 385 patients (39.9%), while the remaining 580 patients demonstrated treatment‐responsive LNs. Among the LN‐positive patients, 224 patients (38.62%) had a single LN metastasis, and 356 patients (61.38%) had multiple LN metastases, with 210 of 356 patients (59.0%) exhibiting a mixed pattern of LN regression. A total of 150 patients (15.5%) were assigned to LNRG 1, 26 (2.7%) to LNRG 2, 141 (14.6%) to LNRG 3, and 263 (27.3%) to LNRG 4. In the combined LNRG 1–2 group, 58.52% of patients had clinical N+ stage, compared with 94.33% in LNRG 3 and 85.93% in LNRG 4. Similarly, the proportions of advanced ypT stage, ypN stage, ypAJCC stage, and lymphovascular invasion (LVI) differed across LNRG groups (Table 1). Six different PD‐1 inhibitors were administered in this study, and their distributions are shown in Table S1. No significant difference in ordinal LNRG was observed among the different PD‐1 inhibitors (*p* = 0.119). In addition, no significant interaction was found between the PD‐1 inhibitor type and LNRG for DFS (*p* = 0.129) or OS (*p* = 0.864). Figure 2A–D shows the histopathological features of LN regression. As described in Section 4, the regression percentage within each LN was derived from the residual tumor relative to total tumor bed (Figure 2E). For patients with multiple metastatic LNs, the average regression was assessed across all metastatic LNs (Figure 2F).

**FIGURE 2:**
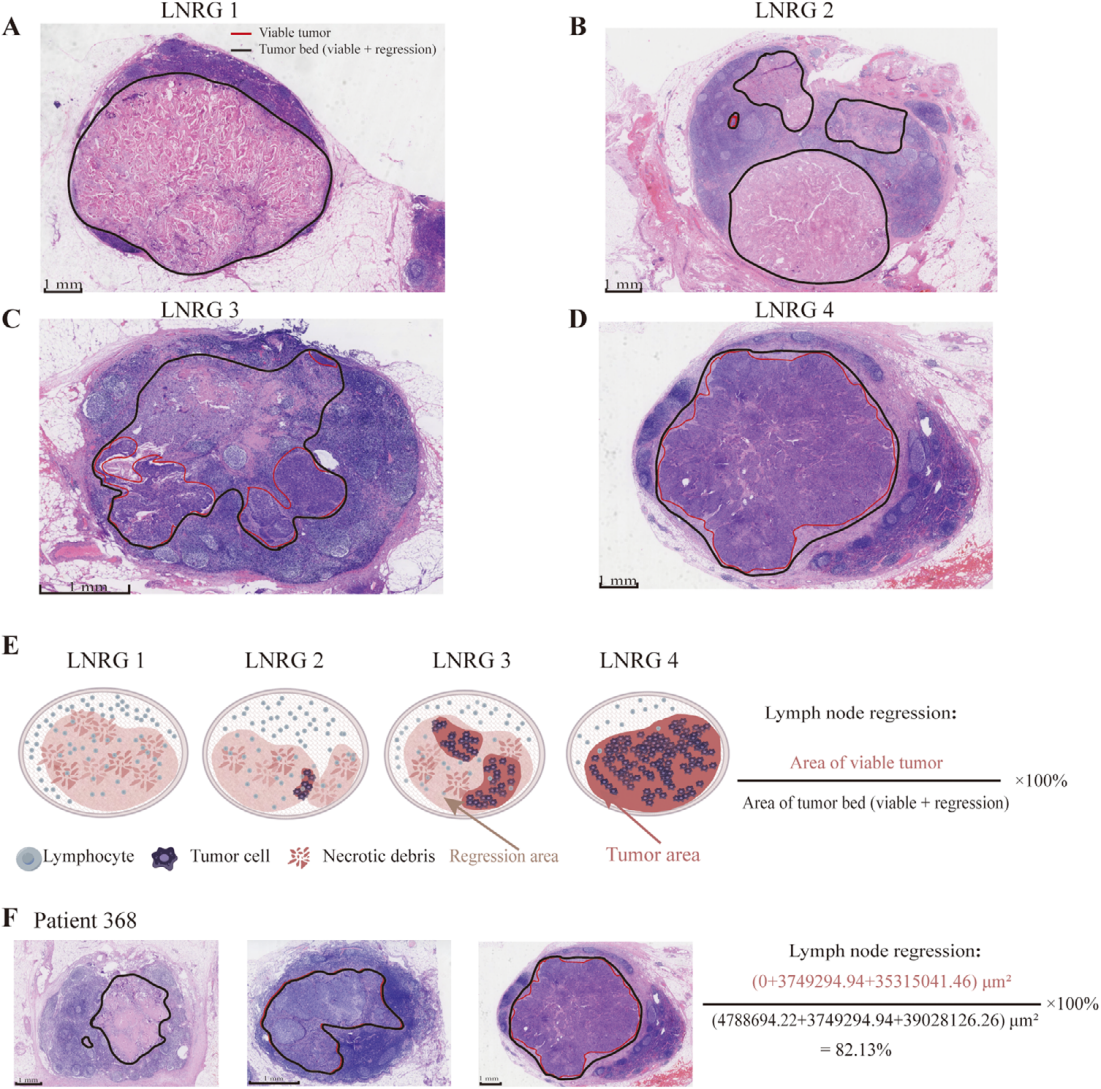
Lymph node regression grade (LNRG) by the novel five‐tier LNRG system. (A–D) Representative hematoxylin and eosin‐stained lymph nodes from LNRG 1–4. LNRG 1, No viable tumor cells observed in the tumor bed area (complete response) (A). LNRG 2, viable tumor cells are observed in < 10% of the tumor bed area (B). LNRG 3, viable tumor cells are observed in 10%–50% of the tumor bed area (C). LNRG 4, viable tumor cells are observed in > 50% of the tumor bed area (D). (E) Schematic illustration of LNRG categorization based on the proportion of viable tumor relative to the tumor bed. (F) Example of average lymph node regression grading in a patient with multiple metastatic lymph nodes. LNRG 0, defined as absence of metastatic lymph nodes, is not shown. LNRG, lymph node regression grade.

The efficacy of the three LNRG definitions, namely average LNRG, optimal‐case LNRG, and worst‐case LNRG, was compared using the 2‐year AUC and Brier score. The average LNRG approach showed the highest discriminatory ability for both DFS and OS. The 2‐year AUCs for DFS were 0.74 (95% CI, 0.71–0.77) for the average LNRG, 0.71 (95% CI, 0.68–0.76) for the worst‐case LNRG, and 0.65 (95% CI, 0.61–0.69) for the optimal‐case LNRG. Similar results were observed for OS, with 2‐year AUCs of 0.67 (95% CI, 0.64–0.72), 0.67 (95% CI, 0.63–0.71), and 0.63 (95% CI, 0.60–0.70), respectively. The average LNRG approach also yielded the lowest 2‐year Brier scores for DFS and OS, indicating better prediction accuracy (Table S2). Therefore, the average LNRG system was selected for the subsequent analyses. In addition, the average LNRG values obtained by conventional microscopy showed high concordance with those calculated using the HALO digital pathology platform (LNRG 0: AC1 = 1, LNRG 1: AC1 = 0.98, LNRG 2: AC1 = 0.99, LNRG 3: AC1 = 0.72, LNRG 4: AC1 = 0.94, and overall AC1 = 0.98; Table S3), supporting the high accuracy of conventional microscopy for average LNRG assessment.

### 2.3 Association Between Lymph Node and Primary Tumor Regression

Based on TRG assessment, 623 patients (64.56%) fell into TRG 1–3 (261 [27.05%] TRG 1, 157 [16.27%] TRG 2, and 205 [21.24%] TRG 3), and 342 (35.44%) patients fell into TRG 4. The cross‐distribution of LNRG and TRG categories was observed in the whole cohort (Figure S1A,B and Table S4). Discordance between the PT and LNRG was identified in 700 of 965 patients (72.54%). Patients with LNRG 1–3 showed a higher rate of PT regression (TRG 1–3) compared with those with LNRG 4: 78.41% of LNRG 1–2 patients and 55.32% of LNRG 3 patients achieved TRG 1–3, whereas only 44.11% of LNRG 4 patients did so (*p* < 0.001). However, a discordance between PT and LN regression was observed: 101 (17.41%) patients with LNRG 1–3 had a PT regression grade of TRG 4, while 116 (20.00%) showed good PT regression (TRG 1–3) but had an LNRG of 4. In adition, a trend toward a higher pathological complete response (TRG 1) rate was observed in the LNRG 1–2 group than in the LNRG 0 group (43.75% vs. 35.84%; *p* = 0.074).

### 2.4 Prognostic Significance of LNRG in NCIT

The median follow‐up time of the entire group was 43.5 (IQR 35.3–54.0) months. We first evaluated the prognostic significance of the five LNRG categories. Kaplan–Meier analysis showed that the 2‐year DFS rates were 73.23%, 89.80%, 80.13%, 67.40%, and 30.41% for LNRG 0, LNRG 1, LNRG 2, LNRG 3, and LNRG 4, respectively. The corresponding 2‐year OS rates were 84.31%, 93.18%, 92.00%, 84.00%, and 63.15%, respectively. Compared with LNRG 1, LNRG 0 was associated with inferior DFS (HR, 2.42; 95% CI, 1.57–3.72) and OS (HR, 2.17; 95% CI, 1.32–3.57; Figure S2). In contrast, LNRG 1 and LNRG 2 showed no statistically significant differences in survival, with comparable DFS (*p* = 0.300) and OS (*p* = 0.559) (Figure S2). Given the limited number of patients in the LNRG 2 subgroup and the absence of a statistically significant survival difference between the LNRG 1 and LNRG 2 groups, we further compared the prognostic performance of the merged LNRG 1–2 model with the unmerged LNRG (LNRG 1 and LNRG 2). Sensitivity analyses showed that maintaining LNRG 1 and LNRG 2 as separate categories did not improve predictive performance compared with the merged LNRG 1–2 model, as indicated by similar 2‐year AUCs and Brier scores for both DFS and OS (Table S5). Therefore, LNRG 1 and LNRG 2 were combined as LNRG 1–2 for subsequent analyses.

Survival analysis demonstrated that patients with LNRG 0, LNRG 3, and LNRG 4 had significantly worse DFS than those with LNRG 1–2. The 2‐year DFS rate was 88.39% for LNRG 1–2, compared with 73.23% for LNRG 0 (HR, 2.24; 95% CI, 1.51–3.32), 67.40% for LNRG 3 (HR, 2.92; 95% CI, 1.89–4.51), and 30.41% for LNRG 4 (HR, 8.03; 95% CI, 5.49–11.76; Figure 3A). Similar results were observed for OS (Figure 3B). The 2‐year OS rate was 93.01% for LNRG 1–2, compared with 84.31% for LNRG 0 (HR, 2.28; 95% CI, 1.42–3.68), 84.00% for LNRG 3 (HR, 2.52; 95% CI, 1.47–4.33), and 63.15% for LNRG 4 (HR, 6.19; 95% CI, 3.89–9.85) (Figure 3B). These findings were further confirmed by Cox regression survival analysis after adjusting for the confounding factors (Table S6). Stratified analyses were performed to evaluate the impact of LNRG on survival. Patients were first stratified by pathological factors (TRG, ypT, and ypN), and then further stratified according to the respective grades for each factor. Across all subgroups, LNRG retained prognostic significance consistent with that observed in the overall study population for both DFS and OS (Figure 3C,D). Finally, because patients with LNRG 0 encompassed various TRG stages (Table S4), we further analyzed survival within these subgroups. The results revealed that patients with LNRG 0 combined with TRG 3–4 (non‐major pathological response [non‐MPR]) had lower 2‐year DFS (63.79% vs. 81.24%; HR 2.26; 95% CI: 1.58–3.24) and lower 2‐year OS (79.23% vs. 88.67%; HR 2.38; 95% CI: 1.53–3.68), compared to those with LNRG 0 and achieved TRG 1–2 (MPR) (Figure 3E,F).

**FIGURE 3:**
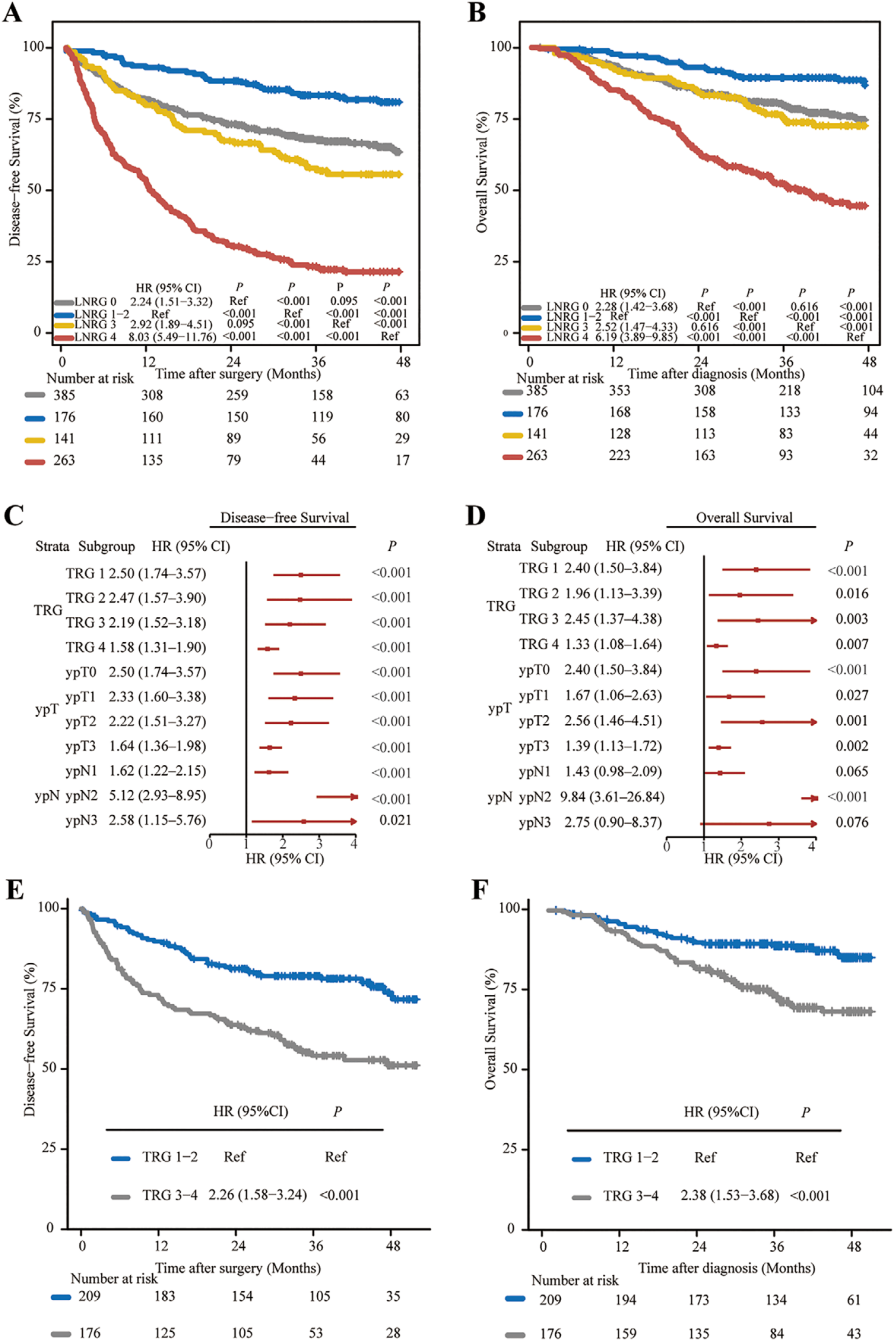
Kaplan–Meier analyses estimate disease‐free survival (DFS) and overall survival (OS) for patients stratified by lymph node regression grade (LNRG). (A, B) Kaplan–Meier survival curves evaluating LNRG for DFS (A) and OS (B). (C, D) Forest plot showing the prognostic value of LNRG for DFS (C) and OS (D) in patients stratified by tumor regression grade (TRG), ypT stage, and ypN stage. *p*‐value, difference among subgroups. (E, F) Kaplan–Meier survival curves evaluating TRG for DFS (E) and OS (F) in the LNRG 0 group. CI, confidence interval; HR, hazard ratio; LNRG, lymph node regression grade; TRG, tumor regression grade.

To further evaluate the comparative prognostic utility of LNRG, we performed NRI and IDI analyses using 2‐year DFS and OS risk estimates. For DFS, LNRG demonstrated improved risk reclassification compared with ypN, with an NRI of 0.24 (95% CI, 0.08–0.37; *p* = 0.020) and an IDI of 0.050 (95% CI, 0.01–0.09; *p* = 0.004). Compared with the TRG, the LNRG also improved the 2‐year DFS risk reclassification, with an NRI of 0.39 (95% CI, 0.08–0.53; *p* = 0.016), while the IDI showed a borderline improvement (0.05; 95% CI, −0.00 to 0.11; *p* = 0.068). For OS, LNRG showed a trend toward improved risk reclassification compared with ypN and TRG, but did not consistently reach statistical significance (Table S7).

### 2.5 Construction and Validation of RSF Model and Online Platform

The entire cohort (*n* = 965) was divided into a training set (*n* = 612, Tianjin Medical University Cancer Center) and a validation set (*n* = 353; the Fourth Hospital of Hebei Medical University and Shanxi Cancer Hospital). Most baseline and pathological characteristics, including age, gender, smoking history, clinical and pathological stage, and TRG, differed between the training and validation cohorts. In contrast, the distribution of LNRGs was comparable between the two cohorts (Table S8). Variable selection for the RSF model was performed in two Cox regression steps in the training cohort. We first screened 11 candidate clinical and pathological variables via univariate Cox analysis; factors with *p* < 0.05 were carried forward, while age, gender, and tumor location were excluded. Collinearity analysis showed low GVIF^(1/(2×DF))^ values for both TRG and LNRG in the multivariate Cox model (1.11 and 1.48, respectively), indicating no substantial multicollinearity between these two variables (Table S9). All univariate‐significant variables were entered into the multivariate Cox regression analysis. According to our predefined threshold (multivariate *p* < 0.1), four independent pathological predictors, including ypAJCC stage, PNI, TRG, and LNRG, and two clinical predictors, including smoking history and BMI, were retained for RSF model development (Table S10). Among these prognostic factors, the LNRG category demonstrated the highest prognostic discrimination for DFS prediction, as evidenced by the lowest AIC (AIC = 2938.52), the highest likelihood ratio chi‐square (LR *χ* ^2^ = 141.95), and superior predictive performance, with a 2‐year AUC of 0.74 (Table S11) in the training set. Three RSF models were developed and compared according to the predictor category: a clinical model (smoking history and BMI), a pathological model (ypAJCC stage, PNI, TRG and LNRG), and a mixed model incorporating all six predictors. In the training set, the 24‐month DFS AUCs of the clinical, pathological, and mixed models were 0.57, 0.83, and 0.85, respectively. In the validation set, the corresponding AUCs were 0.59, 0.75, and 0.76, respectively. Although the mixed model showed a slightly higher AUC than the pathological model, the improvement was minimal in the validation set (*p* = 0.670). Considering the comparable discrimination performance, model parsimony, and clinical interpretability, the pathological model was selected as the final RSF model. A comprehensive analysis of VIMP identified LNRG as the most significant predictor of recurrence compared to the other three factors in the training set (Figure 4A). Patients who experienced recurrence exhibited consistently higher individual risk scores (84.46 ± 56.66) than those who did not in the training set (28.88 ± 27.47, *p* < 0.001; Figure 4B). The model performance resulted in 2‐year AUC values of 0.83 (95% CI, 0.79–0.86) for the training set and 0.75 (95% CI, 0.70–0.80) for the validation set (Figure 4C,D). Decision curve analysis (DCA) indicated that the RSF model exhibited substantial clinical utility (Figure S3A,B). Furthermore, the calibration curves demonstrated satisfactory concordance between the observed and predicted probabilities for the 2‐year DFS in both the training set (Figure S3C) and the validation sets (Figure S3D).

**FIGURE 4:**
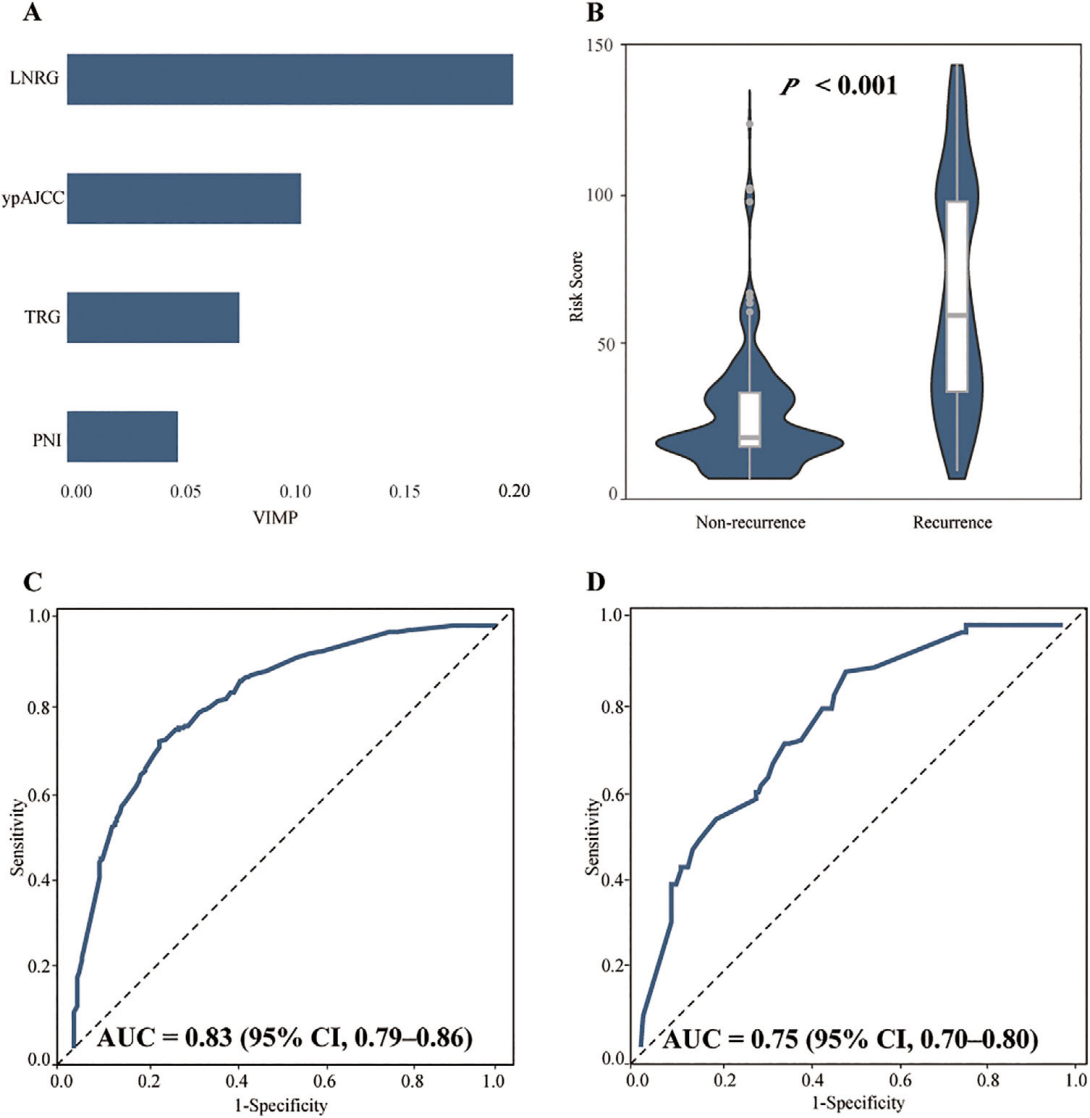
Construction and predictive performance of the random survival forests (RSF) model for recurrence prediction. (A) Variable importance plot for the RSF model in the training cohort. (B) Association between risk score and disease recurrence in the training cohort. (C) Receiver operating characteristic (ROC) curve of the RSF model predicting 2‐year recurrence in the training set. (D) ROC curve of the RSF model predicting 2‐year recurrence in the validation set. AUC, area under the curve; AJCC, American Joint Committee on Cancer; CI, confidence interval; LNRG, lymph node regression grade; PNI, perineural invasion; TRG, tumor regression grade; VIMP, variable importance.

To enhance its clinical applicability, we developed an online platform based on the RSF model to predict the recurrence risk of patients (Figure S4; https://escc1.shinyapps.io/ESCC/). By inputting the actual values of four variables—LNRG, TRG, ypAJCC stage, and PNI—the application can automatically calculate the recurrence‐free probability at different time points for individual patients. This tool assists physicians in conducting prognostic assessments and formulating personalized treatment strategies.

## 3 Discussion

To the best of our knowledge, this is the first large‐scale, multicenter study to propose and validate a novel five‐tier grading system for assessing pathological LNRG following NCIT in patients with ESCC. Our results demonstrate that LNRG serves as an independent indicator of survival, providing prognostic information beyond conventional parameters. Lower stages (LNRG 1–2), which reflect an active treatment response, were associated with superior survival outcomes compared with negative LNs (LNRG 0) and higher stages—an observation not conventionally emphasized in current practice. The LNRG stages did not proportionally align with the TRG. Building on these findings, we developed an RSF model incorporating LNRG, ypAJCC stage, PNI, and TRG, which accurately predicted DFS and can be readily implemented as an online web‐based calculator to facilitate individualized risk stratification.

Recent studies have demonstrated the predictive value of tumor and LN response after NCIT for adjuvant therapy efficacy [11], higher nodal SUVmax predicted residual tumor and worse survival after NCRT [12], and complete LN regression or N downstaging was associated with improved survival after both NCRT and NCT [13, 14]. Multicenter evidence supports the role of LNRG as an independent prognostic determinant of DFS and OS after NCIT. These findings suggest that different treatment modes induce distinct immune mechanisms, leading to variation in the prognostic relevance of LN status. Recent evidence has highlighted that LNs play a crucial role in antitumor immunity by hosting progenitor exhausted CD8^+^ T cells capable of differentiating into effector populations upon PD‐L1 blockade, underscoring LNs as active immunologic sites promoting immunotherapeutic efficacy [7, 15, 16]. These insights into the immunological function of LNs provid a rationale for incorporating LNRG into postoperative evaluations. Notably, our results suggested a discordant regression between PT and LNs. Similarly, in a study of patients undergoing NCT followed by surgery for ESCC, 60% exhibited discordance between PT and LN regression grades [8], and a comparable observation was reported in lung cancer, where the Spearman's rank correlation coefficient between PTs and corresponding LNs was 0.487 [17]. Primary lesions and metastatic LNs differ in cellular composition, and differentiation, genetic alterations, and microenvironmental features, which contribute to distinct biological mechanisms governing their heterogeneous responses to treatment [18, 19]. This evidence demonstrates the complementary value of assessing both LNRG and TRG in prognostic analyses.

Hagi et al. reported that patients with negative LNs had longer recurrence‐free survival than those with ≥ 10% residual tumor in LNs following NCT [8]. In our cohort, we first found that ESCC patients with LNRG 1–2 had a better prognosis than those with LNRG 0. Although LNs in patients with LNRG 1–2 were pathologically involved by tumor, the immune response appeared to be actively promoted in these LNs under NCIT. A trend toward a higher pathological complete response rate in the PT further supports the notion of enhanced systemic antitumor immunity in this subgroup of patients. These results indicated that the sensitivity of PTs and systemic antitumor immune activity may synergistically contribute to treatment outcomes. Conversely, because the LNRG 0 group had no pathological evidence of metastatic nodes, the responsiveness of LNs to NCIT could not be directly assessed. This subgroup may also have included patients with relatively limited immune competence, potentially lowering the DFS and OS of the LNRG 0 group. For such patients, TRG emerged as a more reliable indicator for stratifying DFS and OS, as supported by our data. Taken together, these results highlight that the reinvigoration of antitumor immunity by immunotherapy, particularly within LNs, is a critical determinant of NCIT efficacy.

For LNRG classification, we adopted the five‐tier classification system commonly used in previous studies to ensure reproducibility and clinical applicability [20]. However, standardized criteria for assessing LN regression in patients with multiple metastatic nodes are currently lacking. Among the 965 patients in our cohort, 210 (21.8%) demonstrated a mixed pattern of LN regression involving multiple metastatic nodes. For these patients, relying on the best or the worst LN regression alone may not adequately reflect the actual pathological treatment response. Therefore, we adopted the average ratio of the summed viable tumor area to the summed tumor bed area across all involved LNs as the LNRG value, providing a more comprehensive assessment of the overall nodal response among metastatic sites. Meanwhile, considering the dynamic alterations in regression, we used the total tumor bed as the baseline for determining regression. Our results confirmed that the average LNRG strategy displayed better predictive efficacy than evaluations based on the optimal‐case LNRG (best‐responding node) or worst‐case LNRG (least‐regressed node). Although the HALO platform verified the high reproducibility of the average LNRG system, consistency in the LNRG 3 subgroup (10%–50% residual viable tumor) was relatively lower than that in the remaining subgroups. Similar findings have been reported in a multicenter study by Moore et al., who also reported that the concordance rate of LNRG 3 was only 62.7% [20]. This may be attributed to the histological heterogeneity typically observed in cases with intermediate levels of residual tumor (10%–50%), where residual cancer cells are intermingled with fibrosis, necrosis, and inflammatory components. Developing artificial intelligence for the automated segmentation of tumor regions, areas of previous tumor involvement, and normal tissues may improve the assessment of complex structures, particularly in multiple treated tissues.

Our study has several limitations. First, as this was a retrospective study with inherent selection and information biases, we included three major cancer centers in China and divided the dataset into independent external validation cohorts to minimize bias. Second, because microscopic LN metastases cannot be confirmed before treatment using current diagnostic methods, we relied on established pathological features of tumor bed alterations to identify treatment‐induced changes. Third, further investigation of the immune microenvironmental characteristics of LNs may enhance the interpretability and predictive capacity of the LNRG, which will be addressed in future studies.

In conclusion, this multicenter study established and validated a novel five‐tier LNRG system with encouraging predictive value for NCIT in ESCC. An RSF‐based model integrating LNRG with TRG, PNI, and ypTNM stage accurately predicted recurrence and survival. This LNRG‐based framework offers a more complete view of the treatment response to immunotherapy and may help inform individualized postoperative adjuvant management, pending confirmation in randomized clinical studies.

## 4 Methods and Materials

### 4.1 Study Population

Data for patients with histologically diagnosed ESCC between January 2020 and December 2023 were retrospectively reviewed at Tianjin Medical University Cancer Institute, the Fourth Hospital of Hebei Medical University, and Shanxi Cancer Hospital, which are the major high‐volume EC surgery institutions in eastern and western China, each with a substantial annual caseload of ESCC resections. All three centers have established multidisciplinary teams and use comparable NCIT protocols; these similarities in experience, treatment approach, and pathological evaluation ensured that the study cohorts were representative and allowed for meaningful comparisons across sites. The inclusion criteria were as follows: (1) aged 18–85 years; (2) pretreatment clinical staging of ESCC according to the eighth American Joint Committee on Cancer TNM staging system: cT1b N1–3 M0, or cT2–4a N0–3 M0, corresponding to Stages I to IVa; and (3) Eastern Cooperative Oncology Group (ECOG) performance status score of 0–1. The exclusion criteria included (1) not undergoing esophagectomy; (2) no receipt of neoadjuvant therapy; (3) other types of preoperative treatments (e.g., radiotherapy alone, chemotherapy alone without immunotherapy); and (4) other advanced malignant entities. For model development and validation, patients were grouped by treatment center into a training set (Tianjin Medical University Cancer Center, *n* = 612) and an external validation set (the Fourth Hospital of Hebei Medical University [*n* = 229] and Shanxi Cancer Hospital [*n* = 124]). This study was approved by the Ethics Committee of the Tianjin Medical University Cancer Institute and Hospital (approval number: bc20250579), the Fourth Hospital of Hebei Medical University (approval number: 2026KT417), and Shanxi Cancer Hospital (approval number: KY2026153). Given the retrospective nature of this study, the ethics committees granted a waiver for written informed consent.

### 4.2 Therapeutic Regimen and Follow‐up

Patients received NCIT, defined as at least two cycles of anti‐PD‐1 therapy (one of pembrolizumab, nivolumab, camrelizumab, sintilimab, toripalimab, or tislelizumab; once every three weeks) combined with a platinum‐based doublet (a platinum agent [cisplatin, carboplatin, or nedaplatin] plus paclitaxel or fluorouracil). Surgery was performed 4–8 weeks after the last NCIT cycle. The standard surgical procedure for thoracic ESCC patients is subtotal esophagectomy with two‐field or thre‐field lymphadenectomy. Adjuvant treatment was decided by multidisciplinary discussion based on the pathologic tumor stage and the patient's postoperative status, with recommended regimens of chemotherapy alone, immunotherapy alone, or chemoimmunotherapy.

Patients underwent follow‐up evaluation every 3 months during the first 2 years, and every 6 months thereafter until disease progression, death, or the data cut‐off date. The last follow‐up of all patients was completed in June 2026. Follow‐up assessments included computed tomography (CT) imaging, laboratory tests, and clinical examinations. Disease recurrence was confirmed by definitive radiologic evidence, and histological verification of recurrent lesions was performed in selected cases.

### 4.3 Pathological Evaluation of Primary Tumor and Lymph Node Regression Grades

ESCC specimens were evaluated using a standardized pathology protocol. After surgical specimen isolation, the surgeons immediately completed the LN grouping. The LNs were dissected and labeled separately from the main ESCC specimens.

In initial histological sampling, when no residual tumor or viable tumor cells accounting for less than 10% were observed within the tumor bed. Based on imaging examinations, endoscopic examinations, and tumor appearance before and after neoadjuvant therapy, the tissues suspected to be tumor beds were comprehensively sampled. If the tumor bed measured less than 3 cm, the tumor bed and its surrounding 1 cm area were fully sampled. Conversely, if the tumor bed diameter exceeded 3 cm, at least one section was sampled per 0.5 cm interval. LNs with diameters less than 5 mm were incised along their long axes. For those with a diameter exceeding 5 mm, serial sectioning was performed at 3‐mm intervals. All specimens were routinely fixed in 10% neutral buffered formalin, embedded in paraffin, and stained with hematoxylin and eosin. Two experienced pathologists independently performed histological examinations. They accurately evaluated all slides of each resected specimen, including the tumor's macroscopic features (proximal, distal, and lateral invasion extent) and microscopic features (pathological type, invasion depth, LN status, resection margin status, and pathological response).

LVI was defined as the presence of tumor cells within the lumen of a lymphatic or blood vessel, in contact with endothelial cells, and separated from the vessel wall by a clear gap. The diagnostic criteria for PNI were as follows: (1) cancer cells located outside the nerve sheath but in close proximity to the nerve within the perineural space, with tumor cells encircling more than one‐third of the nerve circumference; and (2) cancer cells infiltrating into the nerve sheath [21].

The percentage of residual viable tumor was calculated as the ratio of residual tumor area to the total tumor bed. For each patient, the overall PT regression was determined by averaging the residual tumor percentages across all slides. Regression was then categorized into four grades according to the Becker system: TRG 1, complete response (no viable tumor); TRG 2, < 10% viable tumor; TRG 3, 10%–50% viable tumor; and TRG 4, > 50% viable tumor [22]. (Figure S1C).

LN regression was histopathologically evaluated based on fibrosis, keratin pearls, and/or multinucleated tumor giant cell reactions present within the LN parenchyma [23]. Necrotic areas or keratin pearls were considered definite histological evidence of tumor regression. Fibrosis with anthracotic pigment deposition was regarded as physiological LN fibrosis and was not considered a regression feature. Pretreatment and post‐treatment CT images were reviewed for LNs showing fibrosis without anthracotic pigment deposition. For patients with multiple metastatic LNs, including those showing complete pathological regression, the total LNRG was defined using three distinct approaches: (1) optimal‐case LNRG (best‐responding node), (2) worst‐case LNRG (least‐regressed node), and (3) average LNRG calculated as the average ratio of the cumulative viable tumor area relative to the cumulative tumor bed area across all metastatic LNs. Continuous LNRG values derived from the three definition methods were further categorized into a five‐tier LNRG system as follows: LNRG 0, without viable tumor or evidence of regression (negative LNs); LNRG 1, complete response (absence of viable tumor in both average and optimal‐case LNRG definitions); LNRG 2, < 10% viable tumor; LNRG 3, 10%–50% viable tumor; and LNRG 4, > 50% viable tumor.

To evaluate the interobserver agreement of the five‐tier LNRG system, two certified pathologists independently assigned LNRG using conventional microscopy or HALO digital pathology platform (Version 3.6.4134, Indica Labs, Albuquerque, NM, USA) on 10,847 LNs from 400 patients (median, 32 LNs for each patient [range, 10–115]). Concordance was measured using Gwet's AC1 Coefficient.

### 4.4 Random Survival Forests Model

The RSF model is used as a regression algorithm based on ensemble learning through decision trees, employing techniques from random forests, such as feature and sample bagging. This approach facilitates a more rapid training process and reduces the estimation bias. The RSF model is particularly suitable for censored survival data, as it modifies the traditional Gini impurity criterion by utilizing log‐rank statistics to determine node splits. This modification aims to maximize the difference between survival curves, as estimated by the Kaplan–Meier method, following a specified cut‐off. In addition, the RSF model can estimate an individual's cumulative hazard function (CHF) by integrating the Nelson–Aalen estimator [24]. In addition, variable importance (VIMP) is assessed by evaluating the decrease in prediction accuracy using out‐of‐bag data, which are not utilized in the construction of each building tree.

### 4.5 Statistical Analysis

The study endpoints were disease‐free survival (DFS) and OS. DFS was defined as the time from the date of surgery to disease recurrence, death from any cause, or last follow‐up. OS was defined as the time from diagnosis to death from any cause or censoring at the last follow‐up.

To identify the optimum categorization of pathological LN response for predicting recurrence, the discriminatory ability of different response classifications was evaluated using the area under the receiver operating characteristic (ROC) curve (AUC) and the Brier score. The net reclassification index (NRI) and integrated discrimination improvement (IDI) were used to compare the prognostic value of LNRG with that of ypN and TRG.

Survival curves were created using the Kaplan–Meier method, with subgroups compared using the log‐rank test. Cox proportional hazards regression was used to assess the prognostic factors for OS and DFS. Multicollinearity among the variables included in the multivariate Cox model was assessed using the generalized variance inflation factor (GVIF^(1/(2×df))^). The factors with *p* < 0.05 in the univariate analysis were included in multivariate analysis to test their association with potential predictors. In the Cox multivariate analysis, variables with *p* < 0.1 were included in the RSF model. The likelihood ratio chi‐square (LR *χ* ^2^) statistics of the corresponding Cox proportional hazards model were applied to assess the homogeneity between different categories. The performance of the models was evaluated both visually and numerically. The AUC, calibration curves, and DCA were used to evaluate the model performance. The model fit was calculated and compared using the corrected Akaike information criterion (AIC) [25]. A lower AIC value or higher LR *χ* ^2^ value implied a more accurate prognostic prediction. AC1 coefficient 0.61–0.80 indicated substantial agreement, and 0.81–1.00 indicated near‐perfect agreement. Statistical analyses reported 95% confidence intervals (95% CI) to quantify the uncertainty of the estimates. Statistical analyses were performed using R version 4.4.2 (https://www.r‐project.org/). Continuous variables were assessed via the Kruskal–Wallis test, while categorical variables were examined using Pearson's *χ* ^2^ test or Fisher's exact test. Interaction terms were added to Cox models to assess whether the prognostic effect of LNRG differed by PD‐1 inhibitor type. Statistical significance was set at *p* < 0.05.

## Author Contributions

J.Z., W.S., J.C., L.R.G., and L.Z. conceptualized the study, developed the methodology, performed the investigations, curated the data, and drafted the manuscript. T.Z., L.G., X.D., X.W., and Y.W. contributed to the methodology, collected the data, and interpreted the data. P.T., H.J., and Q.P. supervised the study, interpreted the data, and revised the manuscript. G.Z., W.Z., and C.Y. conceptualized the study, curated the data, acquired funding, administered the project, supervised the research, and revised the manuscript. All authors have read and approved the final manuscript.

## Funding

This work was supported by the National Natural Science Foundation of China (Grant Numbers 82272733, 82273083, 82503646), State Key Laboratory of Druggability Evaluation and Systematic Translational Medicine (Grant Number QZ23‐9), Tianjin Key Medical Discipline Construction Project (Grant Number TJYXZDXK‐3‐004B), and the Science and Education Cultivation Fund of the National Cancer and Regional Medical Center of Shanxi Provincial Cancer Hospital (Grant Number SD2023018).

## Conflicts of Interest

The authors declare no conflicts of interest.

## Ethics Statement

This study was approved by the Ethics Committee of the Tianjin Medical University Cancer Institute and Hospital (approval number: bc20250579), the Fourth Hospital of Hebei Medical University (approval number: 2026KT417), and Shanxi Cancer Hospital (approval number: KY2026153). Given the retrospective nature of this study and the use of previously collected clinical data, the requirement for written informed consent from the participants was waived by the relevant ethics committees.

## Supporting information




**Supporting Information file 1**: mco271075‐sup‐0001‐SuppMat.docx

## Data Availability

The datasets used during the current study are available from the corresponding author Wencheng Zhang (wczhang@tmu.edu.cn) upon reasonable request.
