# Supplementary material for: Lymph Node Regression Improves Prognostic Stratification in Esophageal Squamous Cell Carcinoma Receiving Neoadjuvant Chemoimmunotherapy: A Multicenter Retrospective Cohort Study
Source: MedComm (2020). 2026 Oct 21;7(11):e71075. doi: 10.1002/mco2.71075 (PMC13686415; doi:10.1002/mco2.71075)
Supplement: Supplementary file 1 [file MCO2-7-e71075-s001.docx]

**Supplementary Materials for**

**Lymph Node Regression Improves Prognostic Stratification in Esophageal Squamous Cell Carcinoma Receiving Neoadjuvant Chemoimmunotherapy: A Multicenter Retrospective Cohort Study**

Jian Zheng, MD;^1^*^#^* Wenbin Shen, MD;^2^*^#^* Jianzhong Cao, MD;^3^*^#^* Linrui Gao, MD;^1^*^#^* Likun Zan, PhD;^4^*^#^* Tian Zhang, MD;^1^ Lei Gong, MD;^5^ Xiaofeng Duan, MD;^6^ Xiaobin Wang, MD;^2^ Yueqin Wu, MD;^4^ Peng Tang, MD;^5^ Hongjing Jiang, MD;^6^ Qingsong Pang, MD;^1^ Gang Zhao, PhD;^7^*^*^* Wencheng Zhang, MD;^1^*^*^* Cihui Yan, PhD^8^*^*^*

^*^Correspondence:

Cihui Yan, PhD.

Email: cihuiyan@tmu.edu.cn

Wencheng Zhang, MD.

Email: wczhang@tmu.edu.cn

Gang Zhao, PhD.

Email: gangzhao@tmu.edu.cn

The file includes:

Figure S1 to S4

Table S1 to S11

**Figure S1.** **Representative histologic image for primary tumor and the association between primary tumor regression and lymph node regression.**

(A) Correlation Between Lymph Node Regression Grade (LNRG) and Tumor Regression Grade (TRG) (n = 965). (B) Pie chart showing the distribution of TRG classifications in different LNRG subgroups. (C) TRG 1, no viable tumor. TRG 2, < 10% viable tumor of tumor bed. TRG 3, 10%–50% viable tumor of tumor bed. TRG 4, > 50% viable tumor of tumor bed.

**Figure S2. Kaplan-Meier survival curves stratified by the five-tier lymph node regression grade (LNRG) classification in the entire cohort.**

(A) Kaplan-Meier survival curves evaluating LNRG for disease-free survival. (B) Kaplan-Meier survival curves evaluating LNRG for overall survival. Abbreviations: CI, confidence interval; HR, hazard ratio; LNRG, lymph node regression grade.

**Figure S3. Predictive performance of the random survival forests (RSF) model for recurrence prediction.**

(A-B) Decision curve analysis (DCA) of the RSF model predicting 2-year recurrence in the training set (A) and validation set (B). (C-D) Calibration curve of the RSF model predicting 2-year recurrence in the training set (C) and validation set (D).

**Figure S4. Screenshot of the online prediction tool based on the random survival fores****ts (RSF) model.**

(A) Prediction interface for Patient X. Postoperative pathology: no response of either the primary tumor (PT) or lymph node (LN); presence of perineural invasion; ypAJCC stage IV. (B) Prediction interface for Patient Y. Postoperative pathology: complete response of both PT and LN metastases; no evidence of perineural invasion. Abbreviations: LNRG, lymph node regression grade; PNI, perineural invasion; TRG, tumor regression grade.

**Table S1. Distribution of LNRG according to PD-1 inhibitor type.**

| PD-1 inhibitors | Total | LNRG 0 | LNRG 1 | LNRG 2 | LNRG 3 | LNRG 4 |
| --- | --- | --- | --- | --- | --- | --- |
| tislelizumab | 263 | 116 (44.11%) | 36 (13.69%) | 9 (3.42%) | 36 (13.69%) | 66 (25.10%) |
| sintilimab | 213 | 76 (35.68%) | 41 (19.25%) | 6 (2.82%) | 27 (12.68%) | 63 (29.58%) |
| pembrolizumab | 184 | 65 (35.33%) | 25 (13.59%) | 3 (1.63%) | 42 (22.83%) | 49 (26.63%) |
| camrelizumab | 208 | 91 (43.75%) | 38 (18.27%) | 4 (1.92%) | 21 (10.10%) | 54 (25.96%) |
| toripalimab | 80 | 28 (35.00%) | 9 (11.25%) | 2 (2.50%) | 13 (16.25%) | 28 (35.00%) |
| nivolumab | 17 | 9 (52.94%) | 1 (5.88%) | 2 (11.76%) | 2 (11.76%) | 3 (17.65%) |

Abbreviations: LNRG, lymph node regression grade.

**Table S2.** **Lymph node regression grading systems and their discriminatory ability.**

| LNRG system | LNRG 0 No.(%) | LNRG 1 No.(%) | LNRG 2 No.(%) | LNRG 3 No.(%) | LNRG 4 No.(%) | 2-year AUC (95%CI) | | 2-year Brier score (95% CI) | |
| --- | --- | --- | --- | --- | --- | --- | --- | --- | --- |
|  |  |  |  |  |  | DFS | OS | DFS | OS |
| Classified by best LNRG | 385 (39.90%) | 267 (27.67%) | 53 (5.49%) | 85 (8.81%) | 175 (18.13%) | 0.65 (0.61–0.69) | 0.63 (0.60–0.70) | 0.21 (0.19–0.22) | 0.16 (0.14–0.17) |
| Classified by worst LNRG | 385 (39.90%) | 150 (15.54%) | 21 (2.18%) | 49 (5.08%) | 360 (37.3%) | 0.71 (0.68–0.76) | 0.67 (0.63–0.71) | 0.20 (0.19–0.21) | 0.16 (0.14–0.17) |
| Classified by average LNRG | 385 (39.90%) | 150 (15.54%) | 26 (2.69%) | 141 (14.61%) | 263 (27.25%) | 0.74 (0.71–0.77) | 0.67 (0.64–0.72) | 0.19 (0.18–0.20) | 0.15 (0.14–0.17) |

Abbreviations: AUC, Area under the curve; CI, confidence interval; DFS, disease-free survival; LNRG, lymph Node regression grading; OS, Overall survival.

**Table S3. Interobserver agreement analysis (n = 400).**

| LNRG | Gwet's AC1 coefficient |
| --- | --- |
|  | （95% CI） |
| LNRG 0 | 1 |
| LNRG 1 | 0.98（0.94–0.99） |
| LNRG 2 | 0.99（0.98–1） |
| LNRG 3 | 0.72（0.59–0.84） |
| LNRG 4 | 0.94（0.88–0.99） |
| Overall agreement | 0.98（0.94–0.99） |

Abbreviations: CI, confidence interval; LNRG, lymph node regression grade.

**Table S4. Correlation Between Lymph Node Regression Grade and Tumor Regression Grade (n = 965).**

| Variable | LNRG0 | LNRG1 | LNRG2 | LNRG3 | LNRG4 | Total |
| --- | --- | --- | --- | --- | --- | --- |
| TRG1 | 138 (14.3%) | 71 (7.4%) | 6 (0.6%) | 19 (2%) | 27 (2.8%) | 261 (27.0%) |
| TRG2 | 71 (7.4%) | 33 (3.4%) | 8 (0.8%) | 20 (2.1%) | 25 (2.6%) | 157 (16.3%) |
| TRG3 | 82 (8.5%) | 17 (1.8%) | 3 (0.3%) | 39 (4.0%) | 64 (6.6%) | 205 (21.2%) |
| TRG4 | 94 (9.7%) | 29 (3.0%) | 9 (0.9%) | 63 (6.5%) | 147 (15.2%) | 342 (35.4%) |
| Total | 385 (39.9%) | 150 (15.5%) | 26 (2.7%) | 141 (14.6%) | 263 (27.3%) | 965 (100.0%) |

Abbreviations: LNRG, lymph Node regression grade; TRG, tumor regression grade.

**Table S5. Sensitivity analysis comparing the prognostic performance of merged and unmerged LNRG classification models.**

| Endpoint | LNRG grouping strategy | 2-year AUC (95% CI) | P for AUC | 2-year Brier score (95% CI) | P for Brier |
| --- | --- | --- | --- | --- | --- |
| DFS | Merged LNRG 1–2 | 0.737 (0.709–0.767) |  | 0.191 (0.177–0.202) |  |
| DFS | Unmerged LNRG | 0.738 (0.709–0.768) | 0.208 | 0.191 (0.176–0.202) | 0.264 |
| OS | Merged LNRG 1–2 | 0.674 (0.639–0.718) |  | 0.154 (0.140–0.167) |  |
| OS | Unmerged LNRG | 0.673 (0.640–0.719) | 0.512 | 0.154 (0.140–0.167) | 0.804 |

The merged LNRG classified patients into four categories: LNRG 0, LNRG 1–2, LNRG 3, and LNRG 4. The unmerged LNRG classified patients into five categories: LNRG 0, LNRG 1, LNRG 2, LNRG 3, and LNRG 4.

Abbreviations: AUC, area under the curve; CI, confidence interval; DFS, disease-free survival; LNRG, lymph node regression grade; OS, overall survival.

**Table S6. Univariable and multivariable cox proportional hazard analysis evaluating disease–free survival and overall** **survival.**

| Variable | Disease–free survival | | | | Overall survival | | | | |
| --- | --- | --- | --- | --- | --- | --- | --- | --- | --- |
|  | Univariate analysis | | Multivariate analysis | | Univariate analysis | | | Multivariate analysis | |
|  | HR (95%CI) | P | HR (95%CI） | P | HR (95%CI） | P | HR (95%CI） | | P |
| LNRG 0 | 2.24（1.51–3.32） | <0.001 | 2.11（1.37–3.25） | <0.001 | 2.28（1.42–3.68） | <0.001 | 1.85（1.12–3.06） | | 0.016 |
| LNRG 1–2 | Ref |  |  |  | Ref |  |  | |  |
| LNRG 3 | 2.92（1.89–4.51） | <0.001 | 1.35（0.58–3.15） | 0.490 | 2.52（1.47–4.33） | <0.001 | 2.80（0.67–11.78） | | 0.159 |
| LNRG 4 | 8.03（5.49–11.76） | <0.001 | 3.44（1.51–7.84） | 0.003 | 6.19（3.89–9.85） | <0.001 | 5.76（1.41–23.59） | | 0.015 |

Abbreviations: CI, confidence interval; HR, hazard ratio; LNRG, lymph node regression grade.

Multivariable analysis was performed after adjusting for confounding factors including age, gender, smoking history, alcohol misuse, body mass index, tumor location, ypAJCC stage, lymphovascular invasion, perineural invasion, and tumor response grade.

**Table S7. NRI and IDI analyses comparing LNRG with ypN and TRG for 2-year DFS and OS.**

| Endpoint | Comparison | NRI (95%CI) | P | IDI (95%CI) | P |
| --- | --- | --- | --- | --- | --- |
| DFS | LNRG vs ypN | 0.24 (0.08, 0.37) | 0.020 | 0.05 (0.01, 0.09) | 0.004 |
| DFS | LNRG vs TRG | 0.39 (0.08, 0.53) | 0.016 | 0.05 (-0.00, 0.11) | 0.068 |
| OS | LNRG vs ypN | 0.18 (0.00, 0.33) | 0.048 | 0.02 (-0.00, 0.05) | 0.152 |
| OS | LNRG vs TRG | 0.32 (-0.20, 0.46) | 0.132 | 0.014 (-0.03, 0.05) | 0.468 |

Abbreviations: CI, confidence interval; DFS, disease-free survival; IDI, integrated discrimination improvemen; LNRG, lymph node regression grade; NRI, net reclassification index; OS, overall survival; TRG, tumor regression grade; ypN, post-neoadjuvant pathological nodal stage.

**Table S8. Baseline and pathological characteristics between training and validation set.**

| **Variable** | **Overall No. (%)** | **Training set**  **No. (%)** | **validation set No. (%)** | *P* |
| --- | --- | --- | --- | --- |
| All | (n = 965) | (n = 612) | (n = 353) |  |
| **Age (year)** |  |  |  | 0.014 |
| ＜65 | 534 (55.34) | 357 (58.33) | 177 (50.14) |  |
| ≥65 | 431 (44.66) | 255 (41.67) | 176 (49.86) |  |
| **Gender** |  |  |  | <0.001 |
| Male | 808 (83.73) | 549 (89.71) | 259 (73.37) |  |
| Female | 157 (16.27) | 63 (10.29) | 94 (26.63) |  |
| **Smoking history** |  |  |  | <0.001 |
| No | 349 (36.17) | 189 (30.88) | 160 (45.33) |  |
| Yes | 616 (63.83) | 423 (69.12) | 193 (54.67) |  |
| **Alcohol misuse** |  |  |  | <0.001 |
| No | 438 (45.39) | 235 (38.40) | 203 (57.51) |  |
| Yes | 527 (54.61) | 377 (61.60) | 150 (42.49) |  |
| **Body mass index** ^a^ |  |  |  | 0.020 |
| <18 | 45 (4.66) | 28 (4.58) | 17 (4.82) |  |
| 18-24 | 617 (63.94) | 410 (66.99) | 207 (58.64) |  |
| >24 | 303 (31.40) | 174 (28.43) | 129 (36.54) |  |
| **Clinical T stage** |  |  |  | <0.001 |
| 1 | 15 (1.55) | 11 (1.80) | 4 (1.13) |  |
| 2 | 49 (5.08) | 15 (2.45) | 34 (9.63) |  |
| 3 | 817 (84.66) | 512 (83.66) | 305 (86.40) |  |
| 4 | 84 (8.70) | 74 (12.09) | 10 (2.83) |  |
| **Clinical N stage** |  |  |  | <0.001 |
| 0 | 293 (30.36) | 94 (15.36) | 199 (56.37) |  |
| 1 | 368 (38.13) | 261 (42.65) | 107 (30.31) |  |
| 2 | 256 (26.53) | 220 (35.95) | 36 (10.20) |  |
| 3 | 48 (4.97) | 37 (6.05) | 11 (3.12) |  |
| **AJCC clinical stage** |  |  |  | <0.001 |
| I | 7 (0.73) | 6 (0.98) | 1 (0.28) |  |
| II | 300 (31.09) | 93 (15.20) | 207 (58.64) |  |
| III | 529 (54.82) | 405 (66.18) | 124 (35.13) |  |
| IV | 129 (13.37) | 108 (17.65) | 21 (5.95) |  |
| **Tumor location** |  |  |  | <0.001 |
| Proximal third | 79 (8.19) | 48 (7.84) | 31 (8.78) |  |
| Middle third | 452 (46.84) | 250 (40.85) | 202 (57.22) |  |
| Distal third | 434 (44.97) | 314 (51.31) | 120 (33.99) |  |
| **ypT stage** |  |  |  | 0.456 |
| 0 | 261 (27.05) | 155 (25.33) | 106 (30.03) |  |
| 1 | 170 (17.62) | 122 (19.93) | 48 (13.60) |  |
| 2 | 182 (18.86) | 129 (21.08) | 53 (15.01) |  |
| 3 | 352 (36.48) | 206 (33.66) | 146 (41.36) |  |
| **ypN stage** |  |  |  | <0.001 |
| 0 | 535 (55.44) | 311 (50.82) | 224 (63.46) |  |
| 1 | 232 (24.04) | 153 (25.00) | 79 (22.38) |  |
| 2 | 144 (14.92) | 105 (17.16) | 39 (11.05) |  |
| 3 | 54 (5.60) | 43 (7.03) | 11 (3.12) |  |
| **AJCC pathologic stage** |  |  |  | 0.002 |
| I | 404 (41.87) | 245 (40.03) | 159 (45.04) |  |
| II | 131 (13.58) | 66 (10.78) | 65 (18.41) |  |
| III | 376 (38.96) | 258 (42.16) | 118 (33.43) |  |
| IV | 54 (5.60) | 43 (7.03) | 11 (3.12) |  |
| **Lymphovascular invasion** |  |  |  | 0.124 |
| Negative | 889 (92.12) | 570 (93.14) | 319 (90.37) |  |
| Positive | 76 (7.88) | 42 (6.86) | 34 (9.63) |  |
| **Perineural invasion** |  |  |  | 0.088 |
| Negative | 929 (96.27) | 594 (97.06) | 335 (94.90) |  |
| Positive | 36 (3.73) | 18 (2.94) | 18 (5.10) |  |
| **TRG** |  |  |  | 0.006 |
| TRG1 | 261 (27.05) | 155 (25.33) | 106 (30.03) |  |
| TRG2 | 157 (16.27) | 129 (21.08) | 28 (7.93) |  |
| TRG3 | 205 (21.24) | 147 (24.02) | 58 (16.43) |  |
| TRG4 | 342 (35.44) | 181 (29.58) | 161 (45.61) |  |
| **LNRG** |  |  |  | 0.723 |
| LNRG0 | 385 (39.90) | 235 (38.40) | 150 (42.49) |  |
| LNRG1-2 | 176 (18.24) | 98 (16.01) | 78 (22.10) |  |
| LNRG3 | 141 (14.61) | 106 (17.32) | 35 (9.92) |  |
| LNRG4 | 263 (27.25) | 173 (28.27) | 90 (25.50) |  |

^a^, Body mass index is calculated as weight in kilograms divided by height in meters squared.

Abbreviations: AJCC, American Joint Committee on Cancer; LNRG, lymph Node regression grade.

**Table S9. Multicollinearity assessment using generalized variance inflation factor for variables included in the multivariate Cox regression model**

| Variable | GVIF ^(1/(2×DF))^ |
| --- | --- |
| Smoking history | 1.28 |
| Alcohol misuse | 1.29 |
| Body mass index ^a^ | 1.01 |
| AJCC pathologic stage | 1.42 |
| Lymphovascular invasion | 1.09 |
| Perineural invasion | 1.04 |
| TRG | 1.11 |
| LNRG | 1.48 |

^a^, Body mass index is calculated as weight in kilograms divided by height in meters squared. Abbreviations: AJCC, American Joint Committee on Cancer; GVIF, generalized variance inflation factor; TRG, tumor regression grade; LNRG, lymph Node regression grade.

**Table S10. Univariable and multivariable cox proportional hazard analysis evaluating disease-free survival in the training cohort.**

| **Variable** | Disease-Free Survival | | | |
| --- | --- | --- | --- | --- |
|  | Univariate Analysis | | Multivariate Analysis | |
|  | **HR（95%CI）** | ***P*** | **HR（95%CI）** | ***P*** |
| **Age (year)** |  |  |  |  |
| ＜65 | Ref |  |  |  |
| ≥65 | 1.04（0.81–1.33） | 0.773 |  |  |
| **Gender** |  |  |  |  |
| Male | Ref |  |  |  |
| Female | 0.77（0.49–1.20） | 0.241 |  |  |
| **Smoking history** |  |  |  |  |
| No | Ref |  |  |  |
| Yes | 1.49（1.12–1.98） | 0.006 | 1.42（1.00–2.02） | 0.053 |
| **Alcohol misuse** |  |  |  |  |
| No | Ref |  |  |  |
| Yes | 1.43（1.10–1.87） | 0.008 | 0.95（0.69–1.32） | 0.781 |
| **Body mass index**^a^ |  |  |  |  |
| <18 | Ref |  |  |  |
| 18-24 | 0.50（0.32–0.80） | 0.004 | 0.62（0.38–0.99） | 0.045 |
| >24 | 0.41（0.25–0.67） | <0.001 | 0.60（0.36–1.01） | 0.056 |
| **Tumor location** |  |  |  |  |
| Proximal third | Ref |  |  |  |
| Middle third | 0.88（0.55–1.41） | 0.589 |  |  |
| Distal third | 0.89（0.56–1.41） | 0.615 |  |  |
| **ypAJCC stage** |  |  |  |  |
| I | Ref |  |  |  |
| II | 2.77（1.76–4.36） | <0.001 | 1.44（0.87–2.38） | 0.152 |
| III | 3.21（2.32–4.44） | <0.001 | 1.57（0.48–5.17） | 0.461 |
| IV | 9.06（5.91–13.90） | <0.001 | 3.04（0.86–10.69） | 0.083 |
| **Lymphovascular invasion** |  |  |  |  |
| Negative | Ref |  |  |  |
| Positive | 2.61（1.79–3.81） | <0.001 | 0.99（0.65–1.51） | 0.967 |
| **Perineural invasion** |  |  |  |  |
| Negative | Ref |  |  |  |
| Positive | 3.28（1.97–5.47） | <0.001 | 2.20（1.24–3.91） | 0.007 |
| **TRG** |  |  |  |  |
| TRG1 | Ref |  |  |  |
| TRG2 | 1.20（0.74–1.96） | 0.454 | 1.08（0.66–1.77） | 0.759 |
| TRG3 | 2.83（1.87–4.28） | <0.001 | 1.56（0.99–2.46） | 0.056 |
| TRG4 | 4.48（3.04–6.60） | <0.001 | 2.37（1.52–3.71） | <0.001 |
| **LNRG** |  |  |  |  |
| LNRG0 | 2.80（1.55–5.07） | <0.001 | 2.72（1.35–5.50） | 0.005 |
| LNRG1-2 | Ref |  |  |  |
| LNRG3 | 3.17（1.68–5.97） | <0.001 | 1.78（0.63–5.04） | 0.276 |
| LNRG4 | 10.51（5.92–18.66） | <0.001 | 5.10（1.86–13.97） | 0.002 |

^a^, Body mass index is calculated as weight in kilograms divided by height in meters squared.

Abbreviations: CI, confidence interval; HR, hazard ratio; LNRG, lymph Node regression grade; TRG, tumor regression grade.

**Table S11. Akaike information criterion analysis, likelihood ratio chi-square and 2-years area under the Curve for the predictors of disease-free survival in the multivariate Cox regression analysis in the train set (n = 612).**

| Variable | LR χ² | AIC | 2-year AUC |
| --- | --- | --- | --- |
| LNRG | 141.95 | 2938.52 | 0.74 |
| ypAJCC | 117.68 | 2962.80 | 0.73 |
| TRG | 90.97 | 2987.50 | 0.71 |
| PNI | 15.19 | 3059.28 | 0.53 |
| BMI | 10.52 | 3065.95 | 0.54 |
| Smoking history | 7.89 | 3066.58 | 0.54 |

Abbreviations: AIC, akaike information criterion; BMI, Body mass index; AJCC, American Joint Committee on Cancer; AUC,area under the curve; LNRG, lymph node regression grade; LR χ2, likelihood ratio chi-square; PNI, perineural invasion; TRG, tumor regression grade.
